# The role of a mechanistic host in maintaining arctic rabies variant distributions: Assessment of functional genetic diversity in Alaskan red fox (*Vulpes vulpes*)

**DOI:** 10.1371/journal.pone.0249176

**Published:** 2021-04-08

**Authors:** Tristan M. Baecklund, Jaycee Morrison, Michael E. Donaldson, Karsten Hueffer, Christopher J. Kyle

**Affiliations:** 1 Environmental and Life Sciences Graduate Program, Trent University, Peterborough, Ontario, Canada; 2 Forensic Science Undergraduate Program, Trent University, Peterborough, Ontario, Canada; 3 Department of Veterinary Medicine, University of Alaska Fairbanks, Fairbanks, Alaska, United States of America; 4 Forensic Science Department, Trent University, Peterborough, Ontario, Canada; 5 Natural Resources DNA Profiling & Forensic Centre, Trent University, Peterborough, Ontario, Canada; UCSI University, MALAYSIA

## Abstract

Populations are exposed to different types and strains of pathogens across heterogeneous landscapes, where local interactions between host and pathogen may present reciprocal selective forces leading to correlated patterns of spatial genetic structure. Understanding these coevolutionary patterns provides insight into mechanisms of disease spread and maintenance. Arctic rabies (AR) is a lethal disease with viral variants that occupy distinct geographic distributions across North America and Europe. Red fox (*Vulpes vulpes)* are a highly susceptible AR host, whose range overlaps both geographically distinct AR strains and regions where AR is absent. It is unclear if genetic structure exists among red fox populations relative to the presence/absence of AR or the spatial distribution of AR variants. Acquiring these data may enhance our understanding of the role of red fox in AR maintenance/spread and inform disease control strategies. Using a genotyping-by-sequencing assay targeting 116 genomic regions of immunogenetic relevance, we screened for sequence variation among red fox populations from Alaska and an outgroup from Ontario, including areas with different AR variants, and regions where the disease was absent. Presumed neutral SNP data from the assay found negligible levels of neutral genetic structure among Alaskan populations. The immunogenetically-associated data identified 30 outlier SNPs supporting weak to moderate genetic structure between regions with and without AR in Alaska. The outliers included SNPs with the potential to cause missense mutations within several toll-like receptor genes that have been associated with AR outcome. In contrast, there was a lack of genetic structure between regions with different AR variants. Combined, we interpret these data to suggest red fox populations respond differently to the presence of AR, but not AR variants. This research increases our understanding of AR dynamics in the Arctic, where host/disease patterns are undergoing flux in a rapidly changing Arctic landscape, including the continued northward expansion of red fox into regions previously predominated by the arctic fox (*Vulpes lagopus*).

## Introduction

Understanding patterns of local adaptation is important to not only enhance insights into how species interact with their environment, but also in clarifying how rapid changes in the suite of selective pressures influence population fitness and persistence [1,2]. When populations undergo divergent selection across heterogeneous landscapes, the potential for local adaptation exists [1,3,4]. The process of local adaptation is not only influenced by selective pressures but also the interplay of gene flow and effective population size (genetic drift) relative to the strength of the selective pressure. Gene flow and genetic drift undermine a population’s ability to locally adapt through either the homogenization of genetic variation or through the random loss of genetic variants in small populations [1,5]. However, if selection is both divergent in nature, and stronger than the combined force of gene flow and genetic drift, local adaptation is likely to occur [1].

Quantitative evidence for local adaptation can be difficult to decipher in natural populations and can be confounded by phenotypic plasticity that allows for the expression of multiple phenotypes from a single genotype [6, 7]. As such, and short of common garden experiments which can be difficult to undertake in natural systems, genetic assessments of the interactions between selection and the demographic forces acting on different populations provide a means to detect patterns indicative of local adaptation. While challenges do exist, genetic signals of locally adapted populations have been identified across a wide range of systems including nonsynonymous gene changes among wolf populations that correlate with precipitation and vegetation patterns [8], and variation in salmonid immune genes associated with the thermal regimes of different waterbodies [9].

Among the array of selective forces that populations are exposed to, infectious diseases often present strong selective pressures capable of enacting rapid and marked population changes as demonstrated by: 1) white-nose syndrome, where disease emergence decimated populations of several species of bats [10,11]; 2) West Nile virus, where over two thirds of crows initially succumbed to the disease [12]; and 3) facial tumors in Tasmanian devils, where populations have experienced 90% declines [13]. In these systems, strong selective sweeps reshaped the genetic diversity of populations through the increased frequency of adaptive traits and a subsequent decrease in the frequency of maladaptive traits [14,15]. The importance of host adaptation in response to disease is further exemplified by chronic wasting disease (CWD) in mule deer, where a genotypic difference conveys resistance to CWD [16]. However, host response to disease is not solely based on the host’s genotype, but also the genetic variants of the disease(s) to which they are exposed, further complicating a population’s interaction with disease based on these coevolutionary patterns [17].

Genetic assessments of local adaptation in response to infectious disease have typically focused on highly polymorphic regions of the major histocompatibility complex (MHC) given associations with antigen binding and overall population health [18–22]. Historically, these studies limit their assessments to a few, if not a single, region (e.g., DRB exon-2) [23–25]. While these studies have provided reasonable assessments of the spatial genetic structure that can arise from local adaptation, the immune system is complex and includes adaptive-, innate-, and intrinsic immunity aspects such that more holistic analyses are required to understand immunogenetic interactions with disease. Several studies have started to use genotyping-by-sequencing (GBS) to explore larger subsets of genetic variation relative to surrounding selective pressures, including disease [e.g., 26–30]. Specifically, Miller et al. [13] developed several GBS arrays, targeting mtDNA and nuclear SNPs, where population structure was indicative of differential responses to the facial tumor disease plaguing Tasmanian devil populations [31].

**Fig 1:**
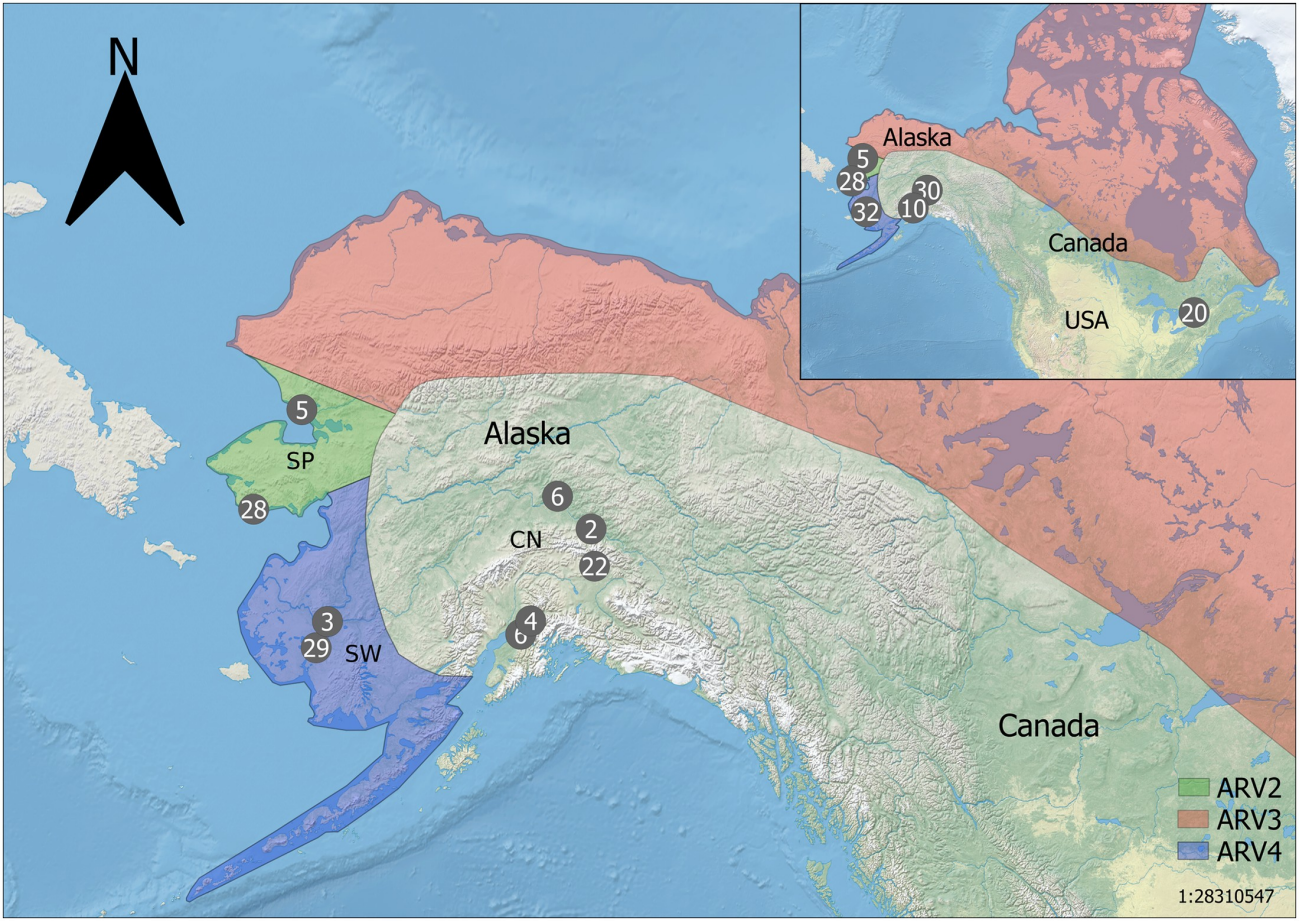
Schematic of red fox samples used in the genotype-by-sequencing assay. Circles indicate sample locations, numbers within circles indicate number of samples within proximity to one another (5mm on the map). CN—Central (South/Interior) Alaska; SP—Seward Peninsula; SW—southwest Alaska; ON—Ontario. Approximate arctic rabies viral variant distributions (AR 2, 3, 4) are depicted by colored regions (see in-figure legend). Insert (top right) shows relative positions of Alaskan red fox samples (n = 105) to those sampled from Ontario (n = 20). The schematic of arctic rabies variant distributions was adapted from Goldsmith et al., 2016 for illustrative purposes only. Made with Natural Earth.

Rabies is a lyssavirus with several strains and subvariants that are normally maintained by a primary/maintenance mammalian host within the orders of Carnivora (e.g., foxes, coyotes, wolves, skunks, and raccoons) and Chiroptera (bats) [32–34]. Rabies is of concern given high mortality rates associated with this disease, the potential for primary hosts to infect domesticated animals and occasionally humans [35], and the fact that the disease can spill over into other reservoir hosts when epizootic [36]. The arctic rabies (AR) strain has a circumpolar distribution that largely coincides with the distribution of its primary host, arctic fox (*Vulpes lagopus*). AR consists of four main viral variants occupying distinct geographical distributions including: variant 2 confined to the Seward Peninsula of Alaska, variant 4 found in southwestern portions of Alaska, and variant 3 which occurs throughout the northern coasts of North America and parts of northern Eurasia [33,34,37] (Fig 1). In contrast, AR variant 1 is isolated to southern Ontario [37], where arctic fox populations are absent and red fox (*Vulpes vulpes*) are presumed to be the maintenance host [38].

In Alaska, three AR variants (2, 3, and 4) circulate in geographically discrete regions where arctic fox and red fox are sympatric in coastal regions. While AR infects both arctic and red foxes, AR is largely absent from the interior of Alaska where only red fox exist [37]. The geographically distinct distribution patterns of AR presence/absence and AR strains in Alaska have led to questions with regards to: i) how this disease is maintained, ii) are there coevolutionary relationships between AR and its hosts that might be expected if patterns of local adaptation exist to explain the geographic restriction of AR variants, and iii) if arctic fox, red fox, or both, serve as maintenance hosts for AR in Alaska. Determining the host status of these fox species is pertinent in that maintenance hosts play different roles in long-term disease maintenance and spread, relative to spillover/reservoir hosts that do not perpetuate disease on broader timescales [36,39,40]. Categorizing disease hosts as either maintenance hosts or spillover hosts can be determined by variable prevalence rates of disease in different host species, as seen with avian influenza in species of waterfowl [41]. However, these correlations sometimes do not provide a holistic understanding of underlying disease dynamics, as in the case of red and arctic fox populations in Alaska, where AR is commonly found in both species; therefore, these patterns may not distinctly differentiate their relative roles with respect to AR [37,41]. Previous research has attempted to use the host genetic structure of both red and arctic fox populations in Alaska, in context of AR strains, to assess the influence of these two hosts on AR disease dynamics [37]. Goldsmith et al. [37] found that population genetic structure of arctic fox correlated with the distribution of the three AR strains as would be expected of a maintenance host and long-term co-evolutionary patterns with AR. The role of red fox was less clear, and data within Goldsmith et al. [37] did not exclude red fox as a maintenance host. Goldsmith et al. [37] did find support for genetic structure among the sampled regions for red fox, where coastal tundra populations clustered together separately from those in the boreal interior [37], aligning with the geographical presence/absence of AR in Alaska. Goldsmith et al. [37] also found evidence of fine-scale geographically isolated genetic clusters, but the levels of admixture among these clusters undermined correlations of the host genetic structure of red fox with AR strains. These findings were not surprising given red fox are widely dispersed carnivores, native to much of the northern hemisphere as a matter of their high dispersal capabilities, their generalist nature, the capacity of the species to exhibit a phenotypic plasticity in response to changes in selective pressures, and historical red fox translocations [42–47]. The lack of correlation of AR strains with low levels of red fox population genetic structure, may also be related to observations that red fox have expanded their distribution northward, coinciding with Arctic warming, a factor postulated to continue to influence and alter AR dynamics [37,48–50].

While the neutral genetic structure of host species can be used to infer relationships with disease maintenance and spread [37,51,52], understanding deeper co-evolutionary patterns between hosts and pathogens requires insight into the variation that exists in genes that interact with the reciprocal selective pressures. To this end and building on the data from Goldsmith et al. [37], Donaldson et al. [53] developed a GBS assay to specifically target 116 immunogenetically relevant regions of the red fox genome. Donaldson et al. [53], tested the assay on a small sample size of red foxes from regions with different AR variants and regions without rabies and found 15 F_ST_-based outlier SNPs that divided the samples into two genetic clusters corresponding to regions with and without AR, similar to the results by Goldsmith et al. [37]. In both studies, inferences on the relationship between host genetic clustering and rabies distributions were undermined by either small sample sizes or assessments on regions of the genome unlikely to show patterns of selection from infectious diseases. However, in these studies, red fox genetic structure was more pronounced in the data from the immunogenetic assay [53] relative to the microsatellite data [37], suggesting that gene flow was not solely responsible for the observed patterns of genetic structure.

Herein, we build on the work of Goldsmith et al. [37] and Donaldson et al. [53], using the same immunogenetic GBS assay, by increasing the number of red fox sampled per location to better assess frequency differences of genetic variants among the sampled locations and to gain further insight into the role of red fox in AR maintenance in Alaska. We also aimed to put the interrelationships of AR and red foxes in Alaska into context by including red fox from Ontario (Canada) as a potential outgroup. Of interest is the contrast between central Alaska and Ontario, as AR variant 1 is solely found in Ontario and is maintained without the presence of arctic fox, where it is detected in red fox and skunk populations [54–56]. It is unclear how the distributions of AR variants are maintained, nor how rapid climate changes occurring in the Arctic may influence rabies disease dynamics such as through the continued northward expansion of red fox (a highly susceptible AR host) into ranges previously predominated by AR’s natural host, the arctic fox [49,50,57–61]. We also aimed to further explore the data from the immunogenetic assay developed by Donaldson et al. [53], by using the well-annotated canine reference genome for enhanced assessments of SNP/gene associations, and by implementing additional SNP outlier tests to account for the inter-variability between methods. We hypothesized that red fox population genetic structure has been shaped by AR variants in Alaska despite high dispersal capability and gene flow found within the species. Therefore, we predicted that immunogenetically relevant genomic regions would demonstrate large shifts in allele frequencies indicative of genetic structure and local adaptation associated with the distribution of AR in Alaska; consistent with the previous findings [53]. This research aims to increase our understanding of how AR is maintained in Alaska, the role of red fox as either a maintenance or spillover host of AR, and the potential role red fox may play as the Arctic continues to experience rapid warming trends which may affect the distribution of AR hosts and its variants.

## Methods

### Sampling, DNA extraction and quantification

Previously collected red fox muscle and spleen tissue samples from Alaska, originally obtained from a variety of independent trappers and organizations, were provided by the University of Alaska Museum of the North (S1 Table). Red fox muscle tissue samples from Ontario (Canada) were obtained from the Ministry of Natural Resources and Forestry (S1 Table). This study required no animal handling or direct sampling from animals (all samples were previously collected), as such ethical approval was not required. Tissue samples were stored in a -80°C freezer and DNA extraction was performed using the DNeasy Blood and Tissue Kit (Qiagen; S1 File). We quantified DNA extractions using the Quant-iT PicoGreen dsDNA Assay Kit (ThermoFisher Scientific). Extracted DNA quality was assessed by ethidium bromide stained 0.8% agarose gel electrophoresis (90 V for 45 minutes) using the HighRanger 1 kbp DNA ladder (300 bp– 10,000 bp; Norgen Biotek) as a reference. After these quantity/quality assessments, a final set of 96 high molecular weight DNA samples suitable for sequencing were processed from four regions across North America: Southwestern Alaska (n = 25), Seward Peninsula (n = 21), Central (South/Interior) Alaska (n = 30), and Renfrew County in Ontario (n = 20).

### Library preparation, sequence capture and high-throughput sequencing

DNA libraries were prepared using the Kapa HyperPlus Kit (Roche) following the SeqCap-EZ HyperCap UGuide v1.0 (Roche) protocol with several modifications to the workflow (S1 File). Pre-capture LM-PCR library quality was assessed utilizing an ethidium bromide stained 2% agarose gel electrophoresis (100 V for 45 minutes).

Equal-molar amounts of each library were combined to form a 1 μg DNA multiplex of the 96 libraries. Target enrichment was performed using the SeqCap EZ Developer Library probe set previously described by our lab [53]. Modifications to the target enrichment included: 2 μl xGen Universal Blockers—TS Mix (Integrated DNA Technologies) instead of the NimbleGen Multiplex Hybridization Enhancing Oligo Pool (Roche), NimbleGen SeqCap EZ Developer Reagent (Roche) was used instead of NimbleGen COT Human DNA (Roche) during hybridization sample preparation, and the hybridization was carried out at 47°C for 20 hours. The target-enriched multiplex was assessed on a bioanalyzer and sequenced on an Illumina MiSeq v3 run using 2x300 bp reads (Advanced Analysis Centre Genomics Facility, University of Guelph). We also obtained previously sequenced data for 29 individuals (S1 Table) [53] from the NCBI Sequence Read Archive (SRP119314) [51] and included that data in subsequent analyses.

### Sequence alignment and variant annotation

Paired-end reads from the 96 newly sequenced individuals, and the 29 previously sequenced samples (total of 125 libraries) were aligned to the canine reference genome (Southwestern Alaska n = 32; Seward Peninsula n = 33; South/Interior Alaska n = 40; Ontario n = 20; Fig 1; S1 Table), utilizing the bwa-mem command in Burrows-Wheeler Aligner v0.7.12 [62]. Sequence alignment metrics were compiled using SAMTOOLS v1.5 [63]. We utilized the Genome Analysis Toolkit (GATK, v4.0.0.0) best practices pipeline and standard hard filtering parameters to perform duplicate sequence removal, SNP/INDEL variant annotation, genotyping, and variant recalibration [64–66]. After these steps, the GATK function SelectVariants was used to filter the obtained VCF file to only contain bi-allelic SNPs.

The original SeqCap EZ Developer Library probe was designed based on limited sequence information from a draft version of the red fox genome [53]; therefore, the positions of all probe targeted areas were first identified within the canine reference genome via BLASTn (S2 Table) which were then complied into a list of on-target intervals. Using these intervals, SNP variants were further categorized as being within coding regions or in intergenic regions (outside coding regions).

Throughout our analyses we addressed several recommendations outlined by other researchers [67] when attempting to identify loci under selection using F_ST_ outlier tests. Specifically, we accounted for possible linkage disequilibrium within the datasets, we implemented a filter for minimum allele frequency (MAF), and we implemented the use of multiple outlier tests in our analyses.

### Analyses of SNPs in intergenic regions

The sub dataset containing SNPs in intergenic regions was filtered using VCFtools, v0.1.13, to retain only biallelic variants with a MAF threshold of 2%, and a maximum missing genotype threshold (per site) of 20%. The remaining SNPs were analyzed using the Ensembl Variant Effect Predictor tool [68,69] to remove any variants that were within 20 kbp from any known transcribed region (protein coding RNA or non-coding RNA) within the reference canine genome. Additionally, these SNPs were pruned for linkage disequilibrium as implemented by the SNPRelate package in R v3.5 [70], and further filtered for physical linkage (only SNPs that were ≥ 100 kbp from one another were retained). Variants that fulfilled these parameters were assumed to not be under selective pressure and were used to assess patterns of neutral population genetic structure. These filtering steps, and subsequent analyses, were also performed on a SNP dataset from intergenic regions that did not include red fox samples from Ontario to test for substructure within Alaska.

Principle component analysis (PCA) and discriminant analyses of principle components (DAPC) were performed in RStudio using the adegenet (v 2.1.1) [71] and ape (v 5.1) [72] packages. The components retained for PCA were those with eigenvalues ≥ 0.1 and cross validation was used to determine the number of retained components based on the root mean squared error (lowest MSE). The optimal number of clusters identified through the data for the DAPC was determined using successive K-means.

Utilizing STRAUTO (v. 1.0) [73], we ran STRUCTURE over several processors concurrently. STRUCTURE analyses were performed using a burn-in length of 50,000 followed by 200,000 iterations for K = 1 through K = 6, with 20 iterations of each K. The ΔK statistic was calculated to determine the number of distinct genetic clusters that were inferred using structure harvester web v0.6.94 [74]. Utilizing CLUMPP 1.1.2 [75], and the LargeKGreedy algorithm (10,000 repeats) individuals were assigned to genetic clusters, the STRUCTURE analyses were combined and visualized using DISTRUCT v1.1 [76].

Power analyses on the presumed neutral SNP datasets were performed using POWSIM v. 4.1 [77]. Simulations were run with Ne = 500 and 5,000, and t = 0, 10, 100, 500, 1,000. 1,000 iterations were performed for each set of conditions, and each run sought to differentiate between the four sampled regions. A Fisher’s exact test was implemented within the program using a Monte Carlo Markov chain approach with the default parameters of 1,000 burn-ins, 100 batches, and 1,000 iterations.

### Analyses of SNPs in coding regions

The sub dataset containing SNPs in coding regions was filtered using VCFtools to retain only biallelic variants with a MAF threshold of 2%, and a maximum missing genotype threshold (per site) of 20%. Outlier testing was performed on this sub dataset using four different packages: PCAdapt [78], OutFLANK [79], Arlequin [80], and Bayescan [81]. Each of these tests identified SNP F_ST_ outliers using an adjusted p-value threshold of ≤ 0.05; detailed parameters implemented for each method are provided as supplement material (S1 File). Outlier tests use different sets of assumptions and caveats, often leading to inconsistencies across packages [79], so we retained any outlier identified by at least one test and compiled these SNPs into a separate VCF file using VCFtools. That dataset was pruned for linkage disequilibrium using the SNPRelate package in R and further filtered for physical linkage by only keeping SNPs > 100 kbp from one another. That sub dataset containing filtered LD-pruned outlier SNPs from coding regions was used to assess the distribution of these variants across our sample design, achieved through the PCA, DAPCs and STRUCTURE analyses described above. Outlier analyses of SNPs from coding regions were also performed with a dataset that did not include red fox samples from Ontario to test for substructure within Alaska.

### Signatures of selection; iHS, XP-EHH, pN/pS

A combined dataset of filtered (MAF and max-missing data) SNPs from both intergenic and coding regions were assessed to determine the integrated haplotype homozygosity score (iHS) and the cross-population extended haplotype homozygosity (XP-EHH) using the REHH package in RStudio (ihh2ihs and ies2xpehh functions respectively) [82,83]. Normalization of p-values for both iHS and XP-EHH are incorporated as part of the REHH package. Normalization is achieved following Gautier and Naves, where p-values are generated through the -log of the Gaussian cumulative distribution function for each statistic [84]. These metrics facilitate a comparison of the integrated extended haplotype homozygosity within a population (iHS) and between populations (XP-EHH) [85,86].

Estimations of the relative ratio of nonsynonymous substitutions to synonymous substitutions (pN/pS ratio) were determined using the output of SnpEff (using the CanFam3.1.99 database) which annotated each SNP within coding regions as synonymous or non-synonymous polymorphisms [87]. Values were calculated per gene following Nei and Gojobori; $pS=\frac{Sd}{S}$ and $pN=\frac{Nd}{N}$ [88]. Where S and N are the number of synonymous and nonsynonymous sites and S_d_ and N_d_ are the total number of synonymous and nonsynonymous polymorphisms [88–90]. Per gene, DnaSP v6 was used to estimate the number of potential nonsynonymous (N) and synonymous sites (S) using the coding sequence for each gene [91]. Ratios > 1 can be indicative of positive selection, whereas ratios < 1 typically infer purifying selection [89].

## Results

### Raw sequence data

The combined dataset (N = 125) of newly (n = 96) and previously (n = 29) sequenced samples had an average of ~315,000 raw reads per library, of which 96.2% mapped to the canine reference genome, ~11.6% reads were filtered per library, and ~58% aligned to targeted regions (~ 65X coverage; S3 Table).

### SNPs in intergenic (off-target) regions

**Fig 2:**
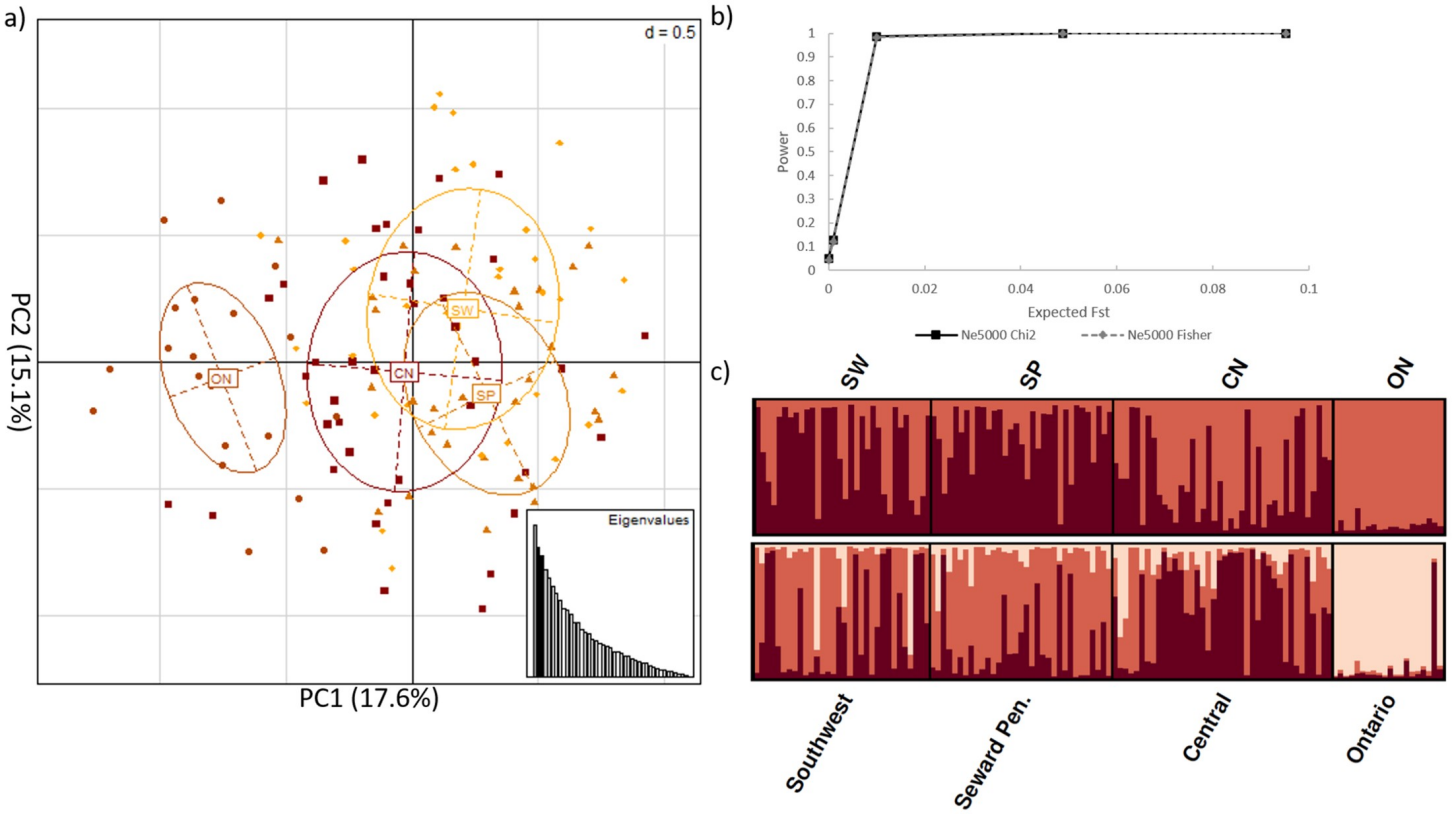
Neutral genetic structure between red fox from Alaska and Ontario. Analyses of the 43 SNPs in intergenic regions after filtering with *Variant Effect Predictor*, a MAF threshold = 2%, and pruning for Linkage disequilibrium; CN = central Alaska; SP = Seward Peninsula; SW = Southwest Alaska; ON = Ontario, Canada **a)** principle component analysis **b)** power analysis results for the estimated Fisher’s exact F_ST_ and Chi^2^ after t = 0, 10, 100, 500, and 1,000 generations and assuming an effective population size of 5,000 **c)** *STRUTURE* analysis of K = 2 and K = 3, where individuals are represented by each bar along the x-axis and assignment to clusters is represented by the y-axis and the different colours.

A dataset of 4,811,979 off-target SNPs was filtered to exclude SNPs < 100 kbp from a coding region and pruned to minimize linkage disequilibrium. This yielded a sub-dataset of 43 SNPs in intergenic regions with an average depth of coverage of 25X that were presumed not to be under selective pressure (S4 Table). We visualized these data using PCA, DAPC and STRUCTURE. Power analyses of the 43 SNPs in intergenic regions indicated a power of ~98% to detect structure at an expected F_ST_ = 0.01 and a power of 100% to detect structure at an expected F_ST_ > 0.05, indicating a high likelihood that if there was population differentiation > 1%, the present dataset had the power to detect it. DAPC and STRUCTURE identified K = 2 as the most likely number of clusters with high levels of gene flow across all analyses (Fig 2). Specifically, Ontario and Alaska formed two distinct genetic clusters (pairwise F_ST_ = 0.035 among genetic clusters), with no clear patterns of genetic structure within the sampled Alaskan regions (Fig 2). Analyses containing only the Alaskan samples provided results similar to those obtained when we analyzed the subset of data containing red fox populations from both Alaska and Ontario; where PCA and DAPC analyses identified no population genetic structure within Alaska, however STRUCTURE results were suggestive of weak patterns of substructure (S1 Fig). This subset of data contained 123 SNPs with an average coverage pers site of ~30X, and an F_ST_ = 0.012 among Alaskan red fox genetic clusters (S4 Table).

### SNPs in protein-coding (on-target) regions

**Fig 3:**
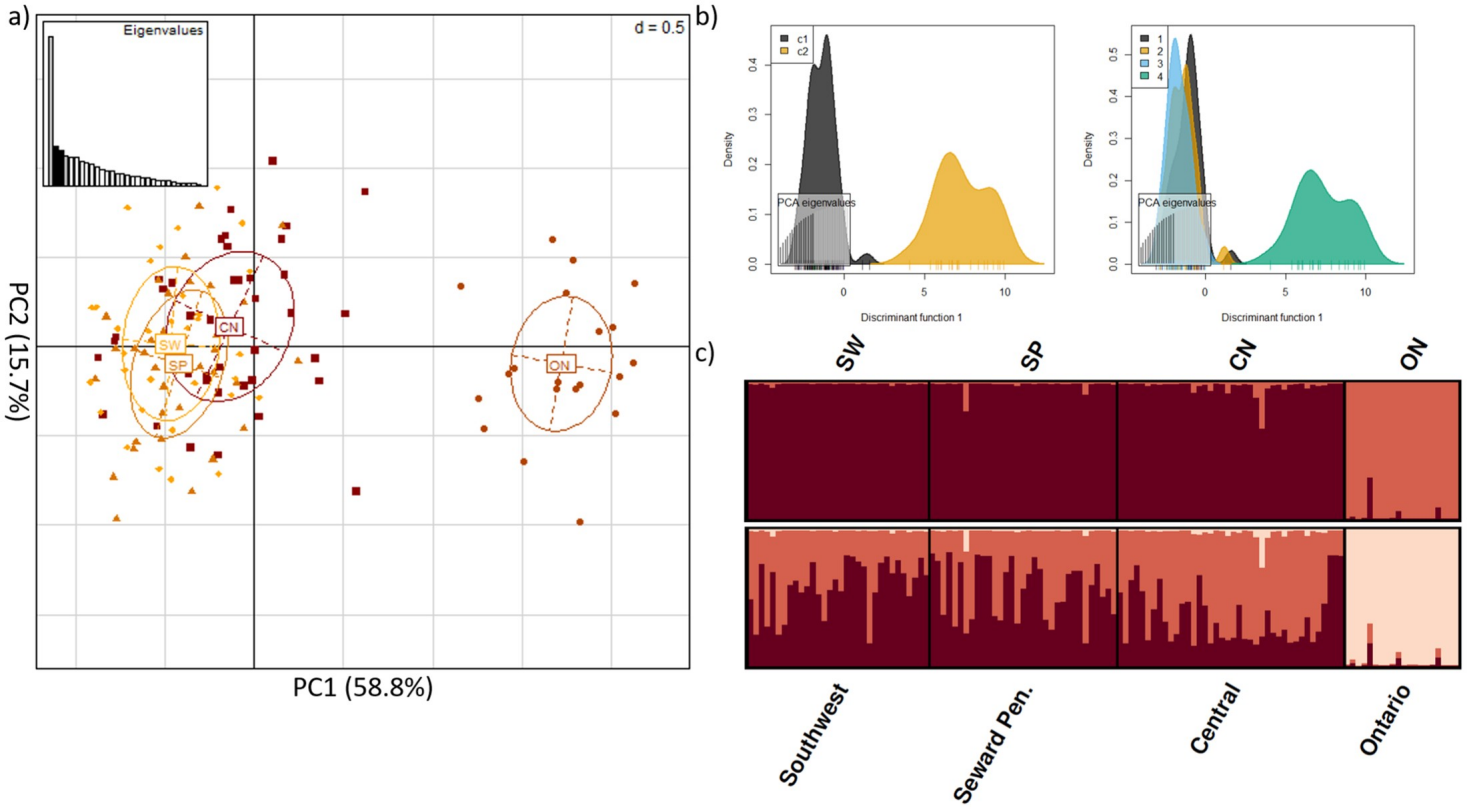
Immunogenetic structure distinguishes between red fox from Alaska and Ontario. Analyses of the 30 outlier SNPs within protein-coding regions after filtering for a minor allele frequency threshold = 2% and pruning for linkage disequilibrium a) principle component analysis b) DAPC of the inferred clustering; c1 = inferred cluster 1, c2 = inferred cluster 2 (left) DAPC of Alaskan (1 = CN; 2 = SP; 3 = SW) vs Ontarian (4) red fox samples (right) c) STRUTURE analyses of K = 2 and K = 3, where individuals are represented by each bar along the x-axis and assignment to clusters is represented by the y-axis and the different colours; CN = central Alaska; SP = Seward Peninsula; SW = Southwest Alaska; ON = Ontario, Canada.

**Fig 4:**
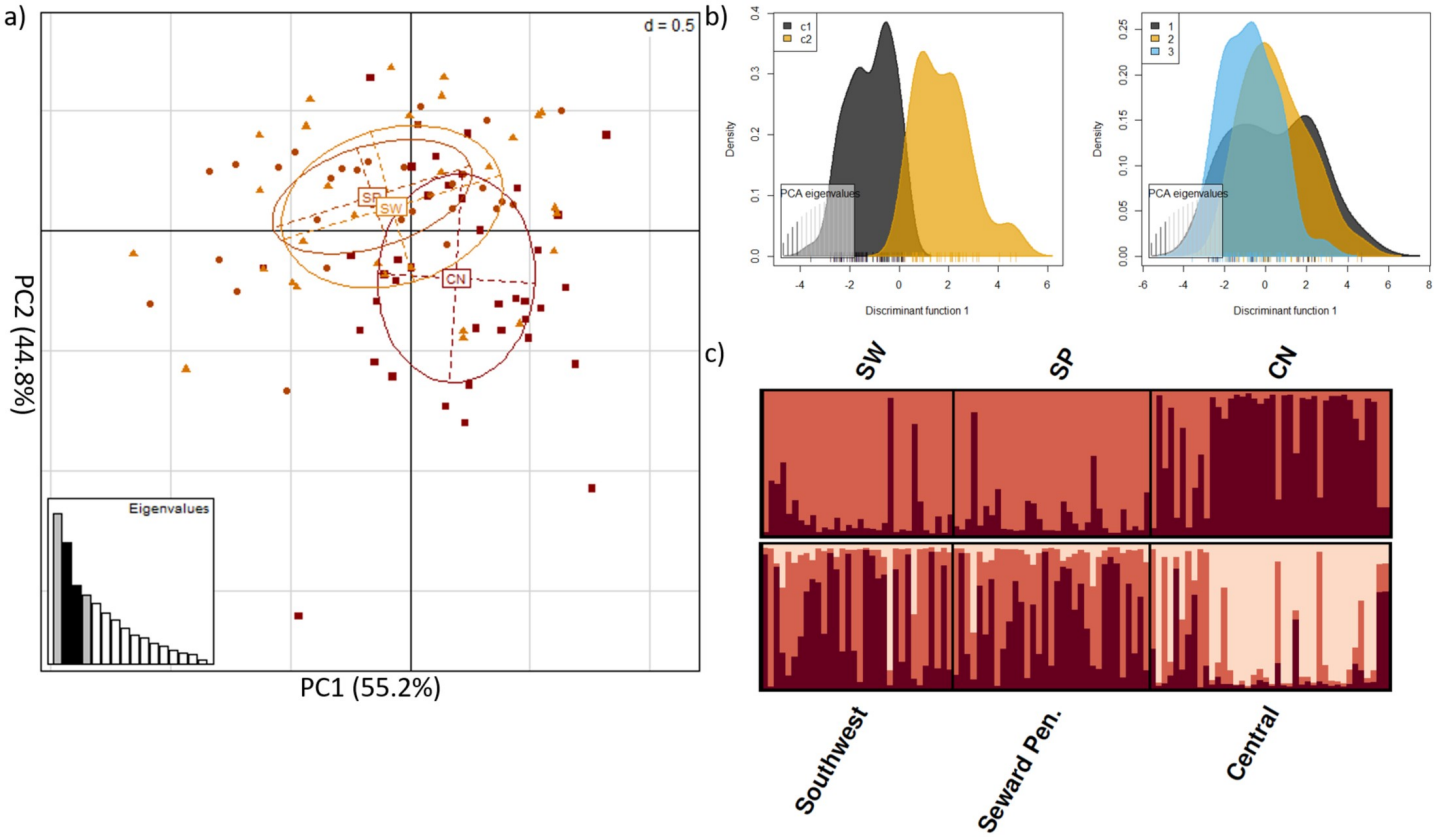
Weak signature of immunogenetic structure among red fox populations within Alaska (not including Ontario). Analyses of the 16 outlier SNPs within protein-coding regions, after filtering for a minor allele frequency threshold = 2%, and pruning for linkage disequilibrium a) principle component analysis b) DAPC of the inferred clustering; c1 = inferred cluster 1, c2 = inferred cluster 2 (left) DAPC of Central (1), Seward Peninsula. (2) and Southwest (3) red fox samples in Alaska (right) c) STRUTURE analysis of K = 2, where individuals are represented by each bar along the x-axis and assignment to clusters is represented by the y-axis and the different colours; CN = central Alaska; SP = Seward Peninsula; SW = Southwest Alaska.

We detected 9,650 SNPs located within on-target intervals. After applying a MAF threshold = 2% a maximum missing data threshold = 20%, and discarding SNPs on the X-chromosome, 2,094 SNPs remained. Only Arlequin and PCAdapt identified outliers, producing a combined sub-dataset of 131 SNPs (S2 Fig). We filtered these 131 SNPs for linkage disequilibrium, producing a sub-dataset of 30 outlier SNPs in protein-coding regions. The majority of these SNPs were found within interleukin, toll-like receptor, and MHC gene families (S5 Table). Two of these SNPs, associated with TLR4 and IL12RB1 genes, were predicted to cause missense mutations that could alter the putative chemical characteristic of the substituent group (S5 Table). We also noted an additional 16 SNPs (from the 131 SNP dataset) removed during linkage disequilibrium filtering were also predicted to cause a missense mutation, potentially altering the underlying chemical property of the respective amino acid. Notably, 12 of these 16 SNPs were found within TLR5 (S5 Table). DAPC and STRUCTURE analyses of the 30 outlier SNPs in protein-coding regions identified K = 2 clusters, which was similar to the results obtained from the off-target SNP analysis, with detectable genetic structure between Alaskan and Ontario foxes (Fig 3). Pairwise assessments found F_ST_ = 0.135 among genetic clusters. Processing these data, obtained from Alaskan foxes exclusively, identified a larger number of outlier SNPs than the dataset that included Ontario foxes (221 versus 131, respectively). Of these 221 outliers, 22 resulted in a missense mutation with the potential to change protein function and one SNP that resulted in a premature stop codon (S5 Table). We filtered the 221 SNPs for linkage disequilibrium and only four of the 22 SNPs with the potential to cause a missense mutation were retained in the sub-dataset that contained 16, filtered and linkage disequilibrium pruned, outlier SNPs (S5 Table). STRUCTURE analyses without the Ontario red fox outgroup suggest weak substructure exists within Alaska, contrasting with both PCA and DAPC analyses that did not reveal distinct population genetic clusters (Fig 4). Pairwise assessments estimated F_ST_ = 0.090 among genetic clusters. Genome wide pairwise F_ST_ of Alaskan red fox are provided as supplementary data (S5 Fig).

### Signatures of selection; iHS, XP-EHH, pN/pS

Potting the p-value of iHS demonstrated few signals of selection for each population. Further analyses of the iHS scores identified one, two, and five outlier candidate regions for the Seward Peninsula, Southwest and Central red fox populations, respectively (S3 Fig). Similarly, XP-EHH analyses demonstrated weak signals of selection between the populations, with the exception of chromosome 18, where the Southwest and Seward Peninsula populations appear to have much closer affinities relative to their comparisons with the Central red fox population (S4 Fig).

From the SnpEff output, we were able to identify missense and synonymous substitutions at 85 of our targeted genes, however, pN/pS calculations were unable to be calculated for any of the three Alaskan red fox populations at 23 of these genes because there were no synonymous and/or missense substitutions at the locations in their respective datasets resulting in division by zero errors or pN values equal to zero (S6 Table). While the majority of the pN/pS ratios were indicative of purifying selection, six genes had pN/pS ratios > 1 in at least one of the populations, suggestive of positive selection (S6 Table). Two of these genes, CCL5 and TLR4 only appeared to be under selection (pN/pS > 1) in the central Alaskan red fox population. The remaining four genes (DLA-12, DLA-88, DLA-DMB, and DLA-DQBC1), appeared to be under selection in all three of the populations.

## Discussion

In this study, we sequenced immunogenetically associated regions of the red fox genome using a GBS assay in context of both variants of arctic rabies (AR) and the presence/absence of the disease. The goal was to further understand the role of red fox in AR maintenance/spread. The GBS assay generated both off- and on-target data. Analysis of these data provided relative assessments of genetic structure that could be attributed to gene flow or associated with local selective pressures. While additional samples, outgroups, and analyses (including the use of a different reference genome) were used in this study, our results were largely consistent with previous investigations [37,53], finding subtle genetic structure between regions with and without the presence of AR, but no evidence of differential selection associated with unique AR variants in among the red fox populations. The latter finding was somewhat unexpected, as it is not clear how distinct AR lineages maintain stable geographic distributions if there is indeed extensive gene flow among red fox, although some evidence implicates the primary AR host, arctic fox, in maintaining these distributions [37]. These data may also suggest that dispersal is more limited among infected red foxes, yet latency of clinical AR symptoms can be weeks and sometimes months, suggesting that limited host dispersal may not be a factor [55,92]. That said, there is no indication that different AR strains show any differences in infectivity or pathogenicity, so not having distinct immunogenetic structure correlated with different AR strains in regions with the disease may then be less surprising. In contrast, the observed levels of neutral genetic structure from the off-target SNP analysis in this study, and previous research [37], are suggestive of high levels of gene flow within all sampled Alaskan regions. Based on the contrasting patterns of neutral and immunogenetic structure, we take these data to suggest that there is a subtle immunogenic response, and potentially a locally adaptive response to AR in red fox populations in Alaska. Further, of the outlier SNPs, several are associated with interleukins and toll-like receptors known to mediate responses to rabies infections [93–97]. Although these data provide further insight into potential mechanisms that control rabies maintenance and spread, additional analyses, such as those resulting from full genome sequencing could reveal different patterns of responses to disease to extend beyond those immunogenetically associated regions.

### Use of off-target data

Targeted sequencing approaches, despite aiming to enrich for certain data, consistently generate undesired (off-target) reads [98]. Explorations of the usefulness of these previously discarded data suggest they possess adequate sequencing coverage and quality for downstream analyses [98–101]. Combining these data with simulation-based assessments, provides a measure of the analytical power of the dataset, and enables confidence to be placed in the interpretation of these data [102–104]. The off-target datasets analyzed herein provide another example of the purposing of otherwise undesirable reads to discern presumed neutral genetic structure as a baseline for the on-target data. Previous red fox research using microsatellite and mtDNA markers [37] found weak genetic clusters that distinguished between the interior of Alaska and coastal regions (F_ST_ = 0.035 among populations), where both clusters displayed extensive admixture with foxes from the northern coast. Our analyses including Ontario red fox, based on presumed neutral off-target SNPs, found no evidence of genetic structuring within Alaska. This contrast in observed structure between studies might reflect the additional power of microsatellite markers, with many alleles per locus, to detect structure, or perhaps the benefit of using certain analytical assumptions which increase the likelihood of identifying subtle patterns of genetic structure as implemented in the work by Goldsmith et al. [37].

The lack of substructure within Alaska using the off-target data, when including Ontario red fox, may be due to stark differences in ancestry between those fox populations from Alaska and Ontario [105–107]. North American red fox occupied several glacial refugia during the last glacial maxima, currently recognized as a Holarctic clade (Western Canada, Alaska, Asia, and European origins) [106] and Nearctic clade (Eastern and Central Canada, the Rocky Mountains, and several montane regions throughout the US) [106]. The recent expansion of red fox into previously unsuitable habitat in the Arctic was thought to be due to the introduction of non-native red fox with European origins, expanding from the Eastern coast of North America across Central US and Canada [105]. However, recent research has shown that expanding red fox populations in the North American arctic tundra are more closely related to the native, boreal, red fox [107]. Therefore, the lack of substructure observed in the off-target SNP dataset may in part be due to the prominent difference in ancestry between fox populations in Alaska and Ontario, thus masking signatures of differentiation between Alaskan populations. The lack of structure observed could also reflect recent population expansions of this species in Alaska, given the potential for genetically similar founders. Off-target analyses of only Alaskan red fox, suggested gene flow exists among the sampled regions despite physiographic features (i.e., mountain ranges) that might be expected to retard gene flow, and supports the supposition that the inclusion of the Ontario outgroup of foxes appeared to mask weak signatures of genetic structure.

### Analysis of SNPs in protein-coding regions

#### Interrelationship between AR and red fox across North America

Methods that identify outliers are prone to varying amounts of type I and II error, which potentially result in inconsistent results between different tests [108]. Analyses of the on-target SNP dataset found only two (Arlequin and PCAdapt) of the four methods identified outliers. While both tests identified a similar number of SNPs, only seven of 131 SNPs were common between the two tests. Most SNPs identified result in synonymous changes (n = 113 SNPs) and are unlikely to alter resulting protein structure and/or function [109]. Of the 18 identified non-synonymous SNPs, a large proportion were associated with TLR5 (n = 12 SNPs), but only the SNPs associated with TLR4 and IL12RB1 were retained in the sub-dataset given our filtering parameters (S5 Table). TLR4 and TLR5 are associated with initiating an inflammatory response by recognizing molecular structures indicative of bacterial infiltration [110–113] whereas IL12RB1 encodes for the transmembrane protein responsible for regulating the response of both IL12 and IL23 [114]. Thus, these genes may have an important role in the species response to disease, especially in the context of rabies, as these gene families have previously been implicated in rabies resistance [93–96]. We also detected outlier SNPs within the protein-coding regions of MHC, interleukins and toll-like receptors (S5 Table). Genetic structure was detected when analyzing the 30 outlier SNPs in protein-coding regions between red fox populations in Alaska and Ontario (F_ST_ = 0.1353). This pattern, relative to the observed weak patterns from the putatively neutral data, is suggestive of local adaptation. The pattern of genetic structure between Alaska and Ontario red fox is interesting in the context of rabies where the AR variant circulating in Southern Ontario (AR variant 1), that is absent from the Arctic, persists without requiring reintroduction from arctic fox, and is phylogenetically distant from the other variants circulating in Alaska [33,34]. While the red fox population was initially implicated in circulating the unique AR variant in Ontario, declines in observations of AR variant 1 among red fox populations, with subsequent increases among skunk populations, lead researchers to test the possibility of a host shift from foxes to skunks [115]. Nadin-Davis and Fehlner-Gardiner found that the variant had accumulated codon changes that coincide with the typical strain of rabies that is found in skunks supporting that this rabies variant may be shifting hosts [115]. The research presented here, combined with this previous work [115], suggest that AR variant 1 may have an increased capacity to locally adapt given the stark differences of the immune response between Ontario red fox populations and red fox populations in Alaska. These differences are made evident from the observed genetic structure (Fig 3), and the potential host shift that is occurring into skunk populations in Ontario.

We acknowledge that correlation does not equal causation, but the identified outliers associated with TLRs and interleukins, coupled with the unique distribution of arctic rabies in Alaska, points towards red fox populations locally adapting to the presence/absence of the disease, but not specific AR variants. Supported by past research demonstrating the involvement of these gene families in rabies mechanisms [93–97], the candidate genes identified herein provide an opportunity to further explore this potential coevolutionary relationship on a larger scale.

#### Interrelationship between AR and red fox in Alaska

When restricting on-target SNP analyses to include only Alaskan red fox, three methods identified outliers (Arlequin, PCAdapt, and OutFLANK). Six of the outliers were identified by at least two programs, demonstrating the variability of program algorithms/assumptions to detect SNP outliers [108]. The outlier SNPs detected by multiple programs were associated with the protein-coding regions of toll-like receptors (TLR5, TLR6) chemokine (C-C motif) ligand 2(CCL2); however, only the SNPs associated with TLR6 and CCL2 were retained in the filtered sub-dataset. TLR6 forms a dimer with TLR2 associated with detecting gram-positive bacteria (S2 Table) [116]. CCL2 is associated with the adaptive immune response by influencing monocyte activity during the inflammatory response (S2 Table) [117]. A large number of the missense variants identified with the potential to change the underlying protein function were associated with the major histocompatibility complex (MHC) and different TLRs. The single nonsense mutation was associated with the protein-coding region of MHC locus DLA-DRB1 that is important in antigen binding. Thus, the respective protein may not be translated properly, which could lead to the loss of antigen recognition and potentially negatively impact the ability of the population to mount an immune response [24].

Estimates of pN/pS ratios of 62 genes offered interesting insights into genes that may be under positive selection between rabies absent areas and those areas where different arctic rabies variants circulate. Specifically, TLR4 appeared to be under positive selection only in the central interior Alaskan red fox population, where rabies is not endemic, indicating that this gene could play a role in preventing the virus from spreading into this region. Further, the four genes under selection in all 3 of the populations (DLA-12, DLA-88, DLA-DMB and DLA-DQBC1) are all components of the MHC. This region plays important roles in antigen recognition and variation within this group of genes is often associated with healthy populations [18–22], as such, it is not surprising to see several MHC gene members under positive selection. Despite these findings, it is important to note that some of the genes studied herein had very few synonymous/nonsynonymous sites which can inflate resulting pN/pS ratios and ultimately lead to potential biasing of these results [89].

We identified genetic structure between red fox from coastal Alaska, where rabies is endemic, and the central interior, where rabies is absent. There was no genetic structure observed in the context of different AR variants encompassed among the sampled regions consistent with previous studies [53]. Despite small sample sizes, a lack of linkage disequilibrium pruning, and the implementation of different outlier testing programs (i.e., LOSITAN), the data presented herein remain consistent with the findings of Donaldson et al. [53]. Additionally, both analyses presented in the current study and those of Donaldson et al. [53] identified SNPs in the protein-coding regions of C3 and ITGAM as outliers. C3 encodes for a protein of the same name, whose derivatives contribute to phagocytosis, an inflammatory response, and relaying signals to T cell-dependant antigens (S2 Table) [118]. ITGAM encodes for *integrin αM*, one of two proteins that bind together to form macrophage antigen 1 which is involved in leukocyte adhesion and migration (S2 Table) [119]. Since SNPs associated with these protein-coding regions have continuously been identified as outliers across studies using several different methods, there is increased support that these genes may be under selective pressure and warrants further investigation.

### Maintenance of arctic rabies variant distributions in Alaska

It has been questioned whether the unique distributions of artic rabies variants in Alaska are influenced by the natural host (arctic fox) and maintained by the red fox. Using microsatellite marker data, Goldsmith et al. [37] demonstrated that the neutral genetic structure of arctic fox appeared consistent with the relative distributions of the rabies variants in the state of Alaska. Goldsmith et al. [37], also found that the neutral genetic structure of red fox only distinguished between those foxes from rabies endemic coastal regions and the rabies absent interior of Alaska [37]. Together, these data were taken to suggest the distributions of AR variants are maintained solely by arctic fox. Data presented in our current study further demonstrate that while the red fox may be a maintenance host for AR in Alaska, the species demonstrates no patterns of genetic structure correlating to the distributions of specific AR variants. In contrast to previous work, however, we find that red fox populations appear to exhibit a weak signature of local adaptation to the presence/absence of AR, as demonstrated by the genetic structure analyses of immunogenetically-relevant loci relative to presumed neutral loci. Furthermore, TLR4 appears to be under positive selection within only the Central red fox population where rabies is not endemic. This gene family has previously been associated with rabies disease mechanisms [96], and the geographic structure of variants of these genes present a potential explanation as to why rabies is not able to reach an endemic status in the central interior of Alaska, although further research would be required to investigate this hypothesis in depth. Overall, these findings are important in context of a warming Arctic, as they suggest that the Alaskan distribution of AR is likely to be unaffected by continued northward expansions of red fox, but rather by a northward retreat of arctic fox from the southern edge of their distribution in the state [50].

### Future steps

Our data suggest red fox populations in Alaska have not undergone differential selection in response to different AR variants based on their unique distributions. It remains plausible, however, that this observation is due to a plastic response of the immune system or that there may be differences in the up/down-regulation of specific genes—hypotheses beyond the scope of the current study. Research utilizing RNA-seq to identify differences in gene expression among foxes exposed to the varying AR variants could have the potential to address some of these alternative hypotheses [120]. Additionally, while the phylogenetic relationship of the AR variants is well documented [33,34], our understanding of whether these genetic differences correspond to underlying differences in pathogenicity or virulence remain unknown. The observed spatial segregation of the AR variants may be caused by founder events with no subsequent gene flow; however, this seems unlikely given the gene flow present within two of the diseases main hosts in Alaska, red and arctic fox, that would homogenize AR variants if they did not have selective differences. Previous research indicates that only arctic fox populations appear to influence the distinct distribution of AR variants [37,53]. This finding, combined with data presented here, suggests selective differences between AR variants do not exist for Alaskan red fox populations. Research should further explore this phenomenon in the natural host of AR, arctic fox, to provide an assessment of genetic differences suggestive of selective differences between AR variants. Given that historical records largely reflect arctic fox have been exposed to AR much longer than red foxes, the likelihood for coevolutionary forces that would result in patterns of differential selection is much greater [55]. Therefore, if such coevolutionary patterns were detected, it would be indicative of differential selection of the different AR variants on arctic fox populations.

## Conclusions

Key to comprehending the ecology and evolution of a species are mechanisms of adaptation [1,3]. When selective pressures are differentially distributed across the landscape, there remains the potential for populations of species to become locally adapted to selective pressures, increasing their fitness within a unique environment [121]. By understanding these interactions between environments and populations, and how they have shaped the genetic structure of populations, we can better inform both wildlife disease- and species-management. Data presented herein suggested that the unique distributions of arctic rabies variants in Alaska have not led to locally adapted populations of red fox, indicating no differential selection between arctic rabies variants. This finding is relevant to wildlife disease management in Alaska and other northern regions as the Arctic continues to warm, likely resulting in range shifts of host species.

## Supporting information

S1 Fig Weak signature of neutral genetic structure among red fox populations within Alaska (not including Ontario). Analyses of the 123 putatively neutral SNPs after filtering for a minor allele frequency threshold = 2% and pruning for linkage disequilibrium **a)** Principle component analysis where 1 = Central, 2 = Seward Peninsula, 3 = Southwest **b)** Power analysis results **c)** STRUCTURE analyses of K = 2 and K = 3 where individuals are represented by each bar along the x-axis and assignment to clusters is represented by the y-axis and the different colours; CN = Central Alaska; SP = Seward Peninsula; SW = Southwest Alaska **d)** DAPC of the inferred clustering of the data **e)** DAPC organized by sampling location (1 = Central Alaska; 2 = Seward Peninsula; 3 = Southwest Alaska).
(TIF)

S2 Fig Schematic of the outlier SNPs before linkage disequilibrium pruning. a) among red fox from Alaska and Ontario; 131 SNP outliers were identified from the sub-dataset that included Ontario red fox. Only PCAdapt and Arlequin identified outliers. b) within Alaska (not including Ontario); 221 SNP outliers were identified from the sub-dataset that did not include Ontario red fox. Only PCAdapt, Arlequin, and OutFLANK identified outliers.
(TIF)

S3 Fig p-value of iHS detects weak signals of selection within three populations of red fox in Alaska. Within population measurements of selective sweeps based on the p-value of the iHS statistic within the Central, Seward Peninsula and Southwest Alaskan red fox populations. Solid grey bars indicate the identified candidate region of iHS outliers for each population.
(TIF)

S4 Fig Assessment of selective sweeps between populations of Alaskan red fox using XP-EHH. Between population measurements of selective sweeps based on the p-value of the calculated XP-EHH statistic between the Central, Seward Peninsula and Southwest Alaskan red fox populations.
(TIF)

S5 Fig Genome wide F_ST_ estimates between 3 populations of red fox in Alaska. Pairwise Weir and Cockerham F_ST_ values between Central, Seward Peninsula, and Southwest Alaskan red fox populations. Identified outliers are highlighted in red and are those represented in the only red fox from Alaska subset in S5 Table.
(TIF)

S1 Table Red fox sample information. Sample identifiers, location, corresponding arctic rabies variant to the area, and accession numbers.
(XLSX)

S2 Table 116 genes probe-bait targets enriched for. Describes in reference to the dog genome (per gene): The transcript and gene ID, position in the genome (chromosome, start/stop base pair), number of exons, and the BLASTp hit description.
(XLSX)

S3 Table GATK filtering results for the 125 red fox samples. Describes (per sample): The number of raw reads, reads passing GATK filters and those reads not passing the GATK filters due to mapping quality, secondary alignments, and duplicate reads.
(XLSX)

S4 Table Filtered off-target SNP sub-datasets. Describes the position and average coverage for each SNP retained in the filtered sub-dataset among red fox populations within Alaska and Ontario, and the filtered sub-dataset among red fox populations exclusively within Alaska. Filtering parameters were a minor allele frequency threshold = 2% and pruning for linkage disequilibrium.
(XLSX)

S5 Table Identified outliers before and after disequilibrium pruning among red fox populations across North America. Describes (per SNP): Location, gene association, and predicted gene function in reference to the dog genome. SNPs are further identified to which sub-dataset they belong; red fox from Alaska and Ontario or red fox only from Alaska: i) identified outlier ii) identified outlier retained after filtering iii) missense SNP with potential to alter protein function and finally if the SNP was identified as an outlier by multiple tests. All BLASTp predicted functions are based upon Canis lupus familiaris unless otherwise specified.
(XLSX)

S6 Table pN/pS ratios for three populations of red fox from Alaska. The ratio of nonsynonymous substitution per nonsynonymous site to synonymous substitutions per synonymous site for 85 genes and three populations of red fox. Those pN/pS ratios >1, suggestive of positive selection, have been bolded. In order for the calculation to be performed, and a pN/pS ratio determined, there must have been at least 1 synonymous and 1 nonsynonymous polymorphism per gene. Per gene, the number of nonsynonymous/synonymous polymorphisms were determined with SnpEff (Cingolani et al. 2012) and the potential nonsynonymous/synonymous sites was estimated from the coding sequence of each gene using DnaSP v6 (Rozas et al. 2017).
(XLSX)

S1 File Supplementary methods. Additional details for methods.
(DOCX)
