# Supplementary figures and images for: The role of a mechanistic host in maintaining arctic rabies variant distributions: Assessment of functional genetic diversity in Alaskan red fox (Vulpes vulpes)
Source: PLoS One. 2021 Apr 8;16(4):e0249176. doi: 10.1371/journal.pone.0249176 (PMC8031376; doi:10.1371/journal.pone.0249176)

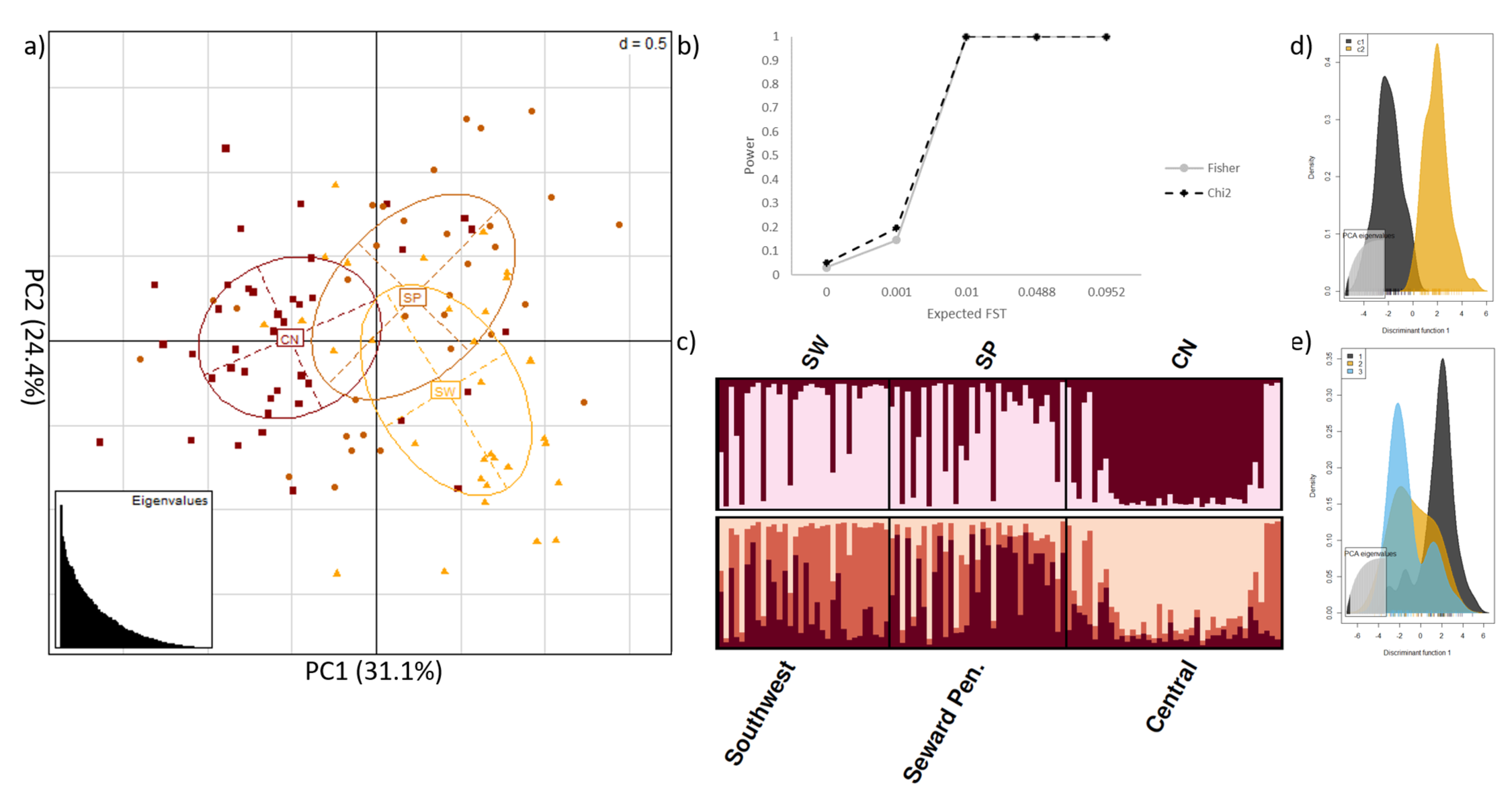

Supplement: S1 Fig — Analyses of the 123 putatively neutral SNPs after filtering for a minor allele frequency threshold = 2% and pruning for linkage disequilibrium a) Principle component analysis where 1 = Central, 2 = Seward Peninsula, 3 = Southwest b) Power analysis results c) STRUCTURE analyses of K = 2 and K = 3 where individuals are represented by each bar along the x-axis and assignment to clusters is represented by the y-axis and the different colours; CN = Central Alaska; SP = Seward Peninsula; SW = Southwest Alaska d) DAPC of the inferred clustering of the data e) DAPC organized by sampling location (1 = Central Alaska; 2 = Seward Peninsula; 3 = Southwest Alaska). (TIF) [file pone.0249176.s001.tif]

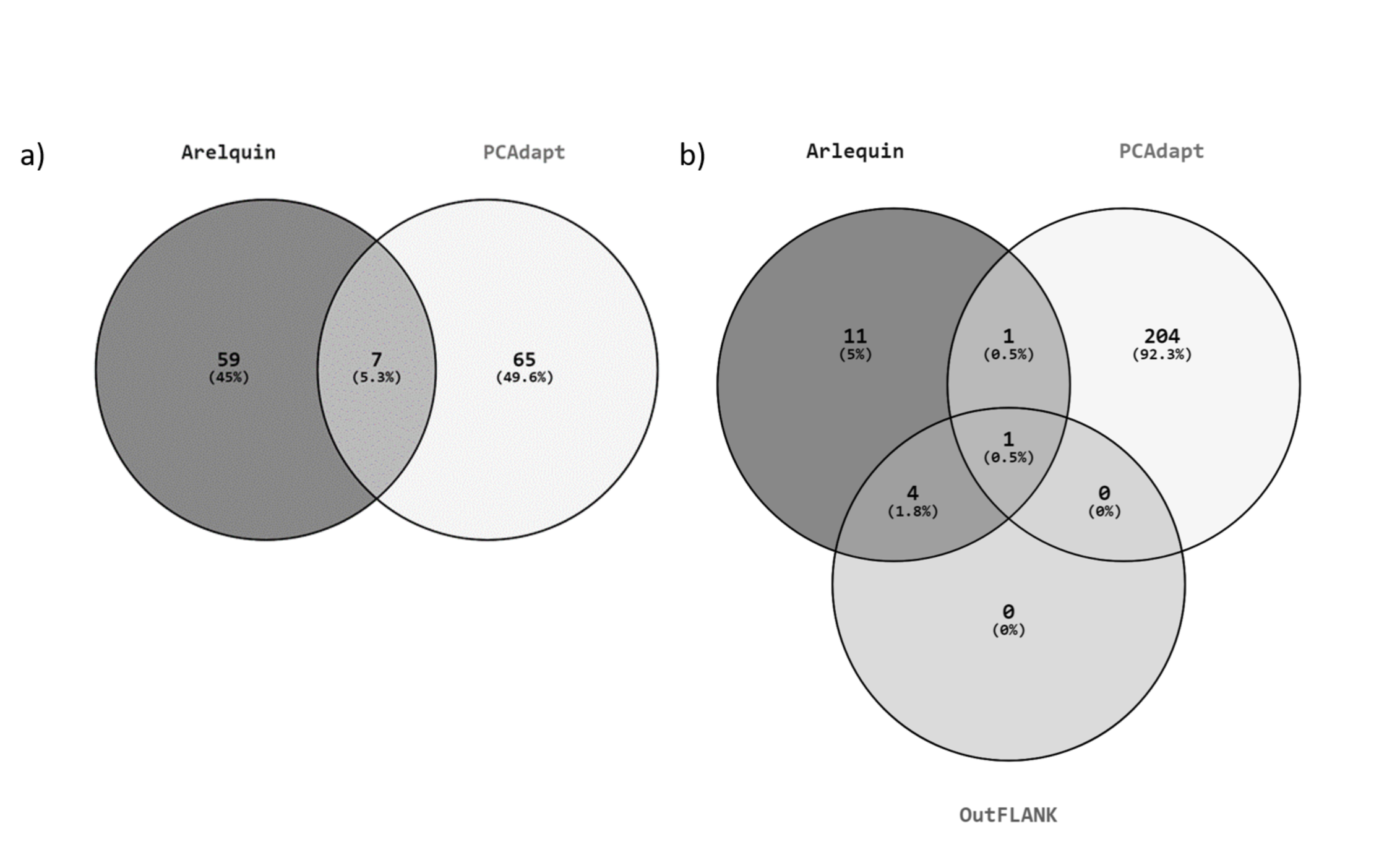

Supplement: S2 Fig — a) among red fox from Alaska and Ontario; 131 SNP outliers were identified from the sub-dataset that included Ontario red fox. Only PCAdapt and Arlequin identified outliers. b) within Alaska (not including Ontario); 221 SNP outliers were identified from the sub-dataset that did not include Ontario red fox. Only PCAdapt, Arlequin, and OutFLANK identified outliers. (TIF) [file pone.0249176.s002.tif]

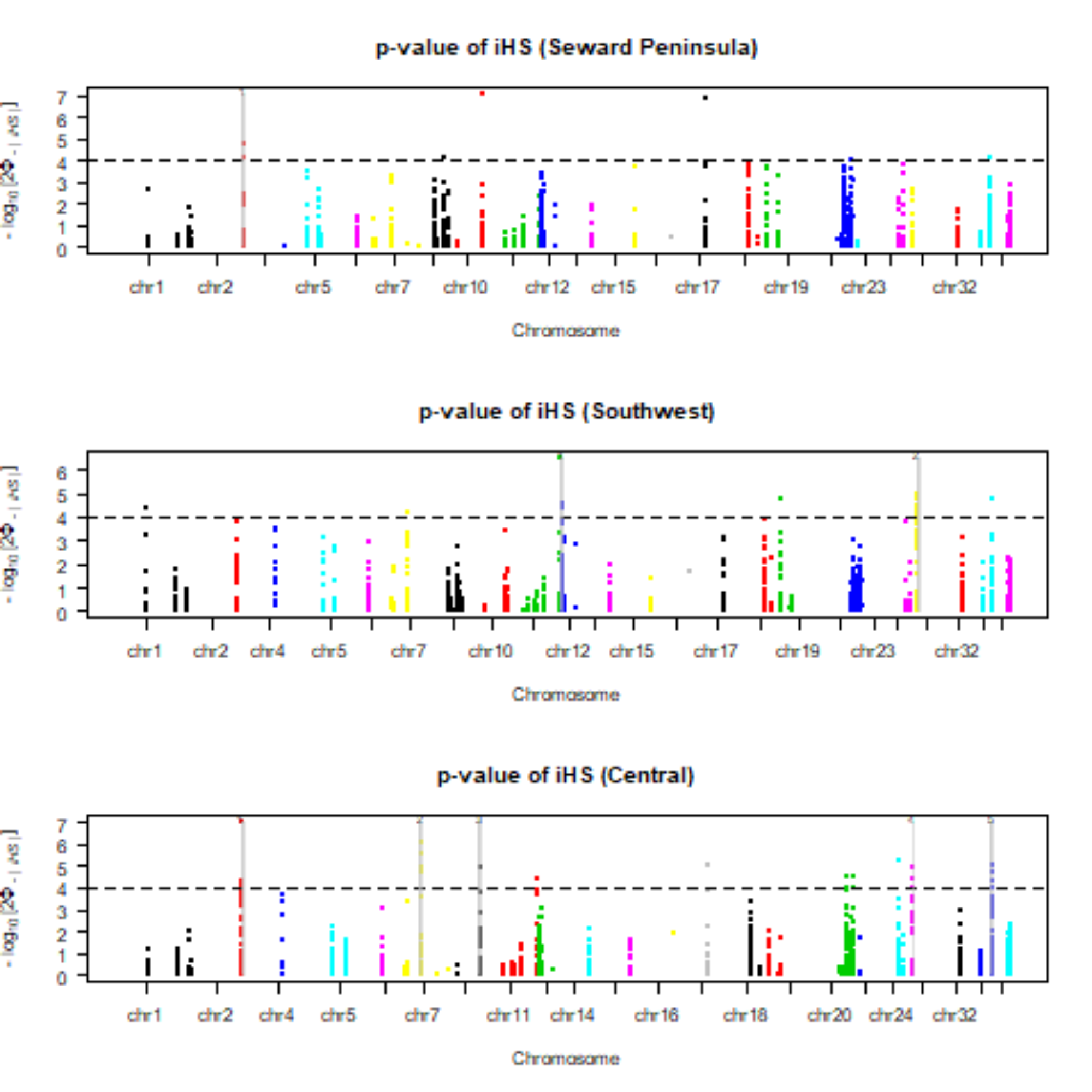

Supplement: S3 Fig — Within population measurements of selective sweeps based on the p-value of the iHS statistic within the Central, Seward Peninsula and Southwest Alaskan red fox populations. Solid grey bars indicate the identified candidate region of iHS outliers for each population. (TIF) [file pone.0249176.s003.tif]

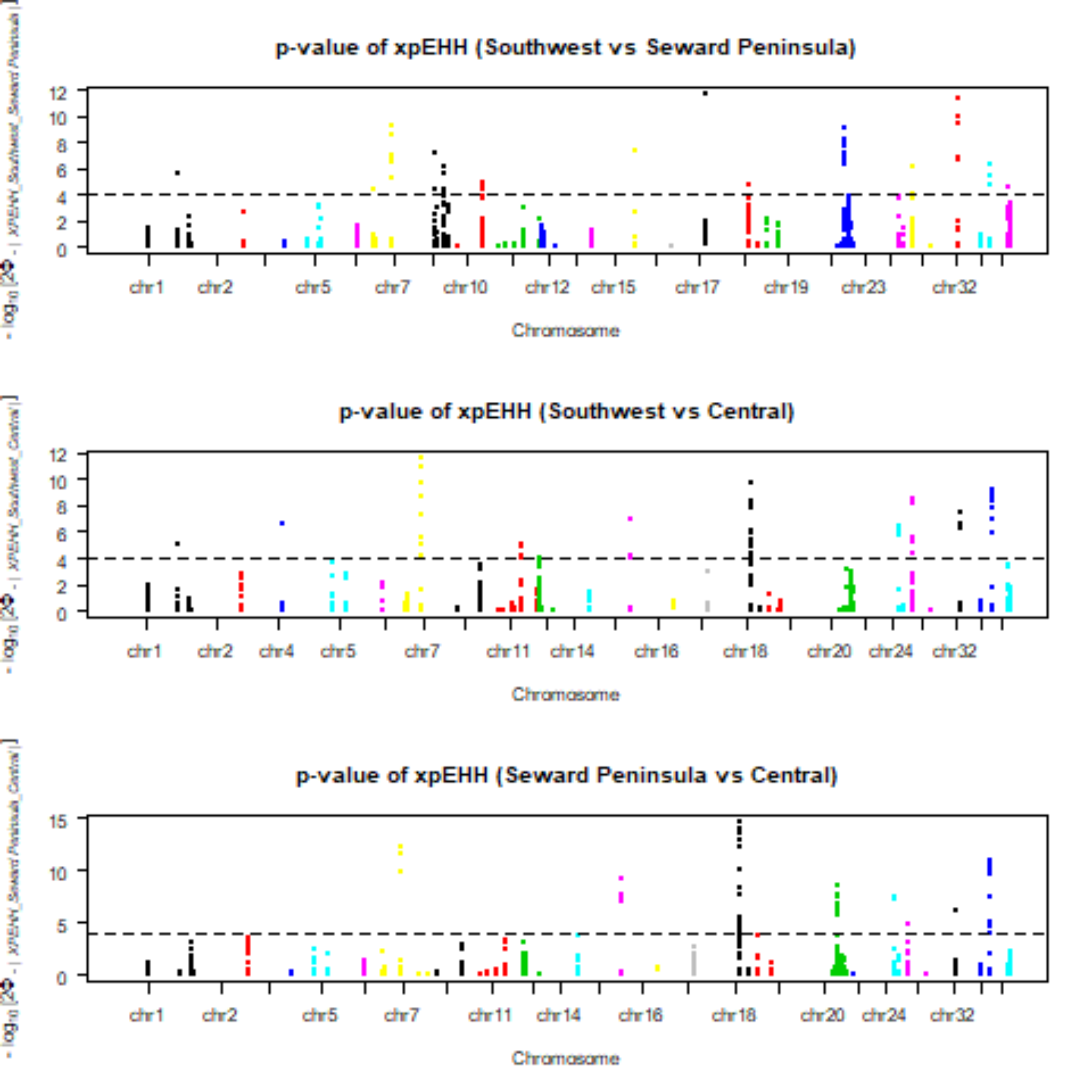

Supplement: S4 Fig — Between population measurements of selective sweeps based on the p-value of the calculated XP-EHH statistic between the Central, Seward Peninsula and Southwest Alaskan red fox populations. (TIF) [file pone.0249176.s004.tif]

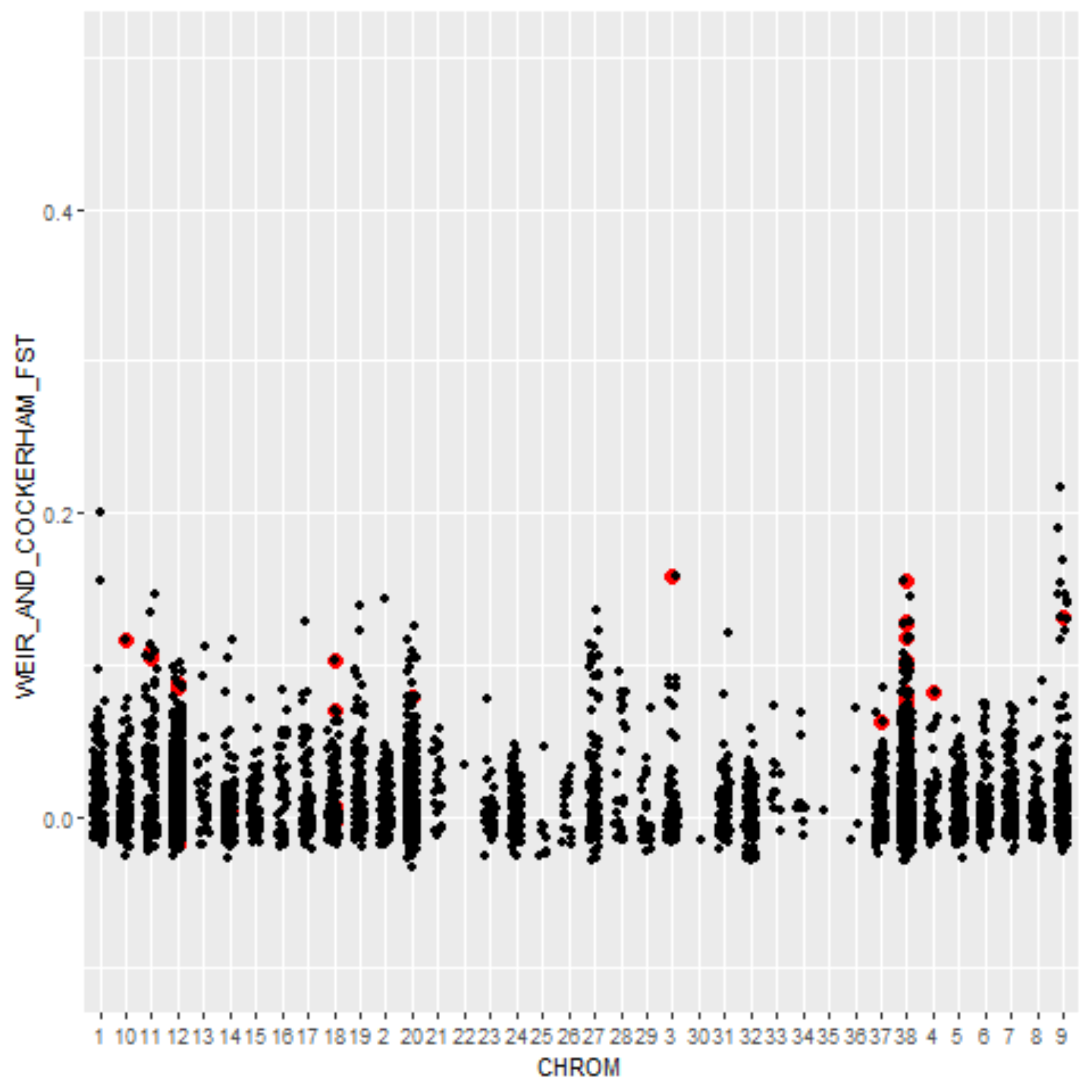

Supplement: S5 Fig — Pairwise Weir and Cockerham FST values between Central, Seward Peninsula, and Southwest Alaskan red fox populations. Identified outliers are highlighted in red and are those represented in the only red fox from Alaska subset in S5 Table. (TIF) [file pone.0249176.s005.tif]
