# Supplementary material for: The role of a mechanistic host in maintaining arctic rabies variant distributions: Assessment of functional genetic diversity in Alaskan red fox (Vulpes vulpes)
Source: PLoS One. 2021 Apr 8;16(4):e0249176. doi: 10.1371/journal.pone.0249176 (PMC8031376; doi:10.1371/journal.pone.0249176)
Supplement: S1 File — Additional details for methods. (DOCX) [file pone.0249176.s012.docx]

# Supplementary Methods

## General Methods

### Sampling, DNA extraction and quantification

Tissue and pelt samples were dissolved in 200 μL 1X lysis buffer (4 M Urea, 0.2 M NaCl, 0.5% n-lauroyl sarcosine, 10 mM Ethylenediaminetetraacetic acid (EDTA), 0.1 M Tris HCl pH 8.0) with the addition of 20 μL proteinase K and incubated at 56°C for two hours (vortex and brief centrifuge every 0.5 hr). During pelt sampling, hair was cut off with a No. 22 sterile Paragon surgical blade, leaving only the follicle and small portions of the cortex remaining in the sample. Samples were extracted using the DNeasy Blood and Tissue Kit (Qiagen) following the standard protocol with the exception that DNA was eluted off the column twice with 30 μL TE buffer (10 mM Tris, 0.1 mM EDTA).

### Library preparation, sequence capture and high-throughput sequencing

Seven cycles were used for the pre-LM PCR as recommended by the manufacturer. Modifications to the DNA library preparation workflow included: PCR-grade water used instead of 10 mM TrisHCl (pH 8.0) for dilutions and elutions, samples were treated with 5 μL conditioning solution included within the HyperPlus Kit during fragmentation, TruSeq HT Dual-Index Adapters (Integrated DNA Technologies) re-suspended in Nuclease Free Duplex Buffer (Integrated DNA technologies) were substituted in place of the SeqCap Adapter Kits A and B (Roche) during adapter ligation, and Illumina P5 and Illumina P7 primers (Integrated DNA technologies) were utilized instead of the Pre LM-PCR Oligos 1 & 2 (Roche) during Pre-Capture LM-PCR.

Individual libraries were quantified with the Quant-iT PicoGreen dsDNA Assay kit (ThermoFisher Scientific), normalized to 4 ng/μL and quantified again using the same method. Target enrichment was performed using the workflows outlined in the text using the specially designed SeqCap EZ Developer Library probe set described in Donaldson et al. (2018), with the following additional modifications: (i) 2 μl xGen Universal Blockers - TS Mix (Integrated DNA Technologies) were used instead of the NimbleGen Multiplex Hybridization Enhancing Oligo Pool (Roche), and (ii) NimbleGen SeqCap EZ Developer Reagent (Roche) was used instead of NimbleGen COT Human DNA (Roche) during hybridization sample preparation where the hybridization was carried out at 47°C for 20 hr. The target-enriched multiplex was assessed on a bioanalyzer and sequenced on a MiSeq v3 run using 2x300 bp reads (Advanced Analysis Centre Genomics Facility, University of Guelph).

## Outlier Testing Parameters

### PCAdapt v4.0.2

Outlier testing was implemented in RStudio using the pcadapt, vcfR, and qvalue packages. K values of 2-4 were used in conjunction with datasets generated denoting the respective number of clusters. Qvaules were assessed with a threshold of 0.05.

### OutFLANK v0.2

Outlier testing was implemented in RStudio using OutFLANK, ggplot2, and vcfR packages. We used a left- and right- trimfraction of 0.05, minimum expected heterozygosity of 0.1, qthreshold of 0.05, and modified the NumberOfSamples to reflect the number of K-clusters being tested.

### Arlequin v3.5.2.2

Detecting loci under selection was achieved using: 20,000 simulations, 100 demes simulated per group, minimum expected Heterozygosity of zero, maximum expected heterozygosity of one, and assessing pairwise differences. Population comparisons were achieved using: 100 permutations, a significance level of 0.05, a gamma *a* value of zero. Pairwise differences were calculated, and distance matrices were computed. Population differentiation metrics were completed using: genotype frequencies, a 100,000 step Markov Chain, 10,000 dememorization steps, and a significance level of 0.05.

### BayeScan v2.1

We implemented our outlier analysis using Bayescan with: a sample size of 5,000, a thinning interval of 10, 20 pilot runs each with a length of 5,000, a burin in of 50,000, and prior odds for the neutral model of 10.

References

1. Luu K, Bazin E, Blum MG. pcadapt: an R package to perform genome scans for selection based on principal component analysis. Molecular ecology resources. 2017 Jan;17(1):67-77.
2. Whitlock MC, Lotterhos KE. Reliable detection of loci responsible for local adaptation: Inference of a null model through trimming the distribution of F ST. The American Naturalist. 2015 Oct 1;186(S1):S24-36.
3. Excoffier L, Lischer HE. Arlequin suite ver 3.5: a new series of programs to perform population genetics analyses under Linux and Windows. Molecular ecology resources. 2010 May;10(3):564-7.
4. Foll M, Gaggiotti O. A genome-scan method to identify selected loci appropriate for both dominant and codominant markers: a Bayesian perspective. Genetics. 2008 Oct 1;180(2):977-93.
